# *Astragalus* Polysaccharides Alleviate Type 2 Diabetic Rats by Reversing the Expressions of Sweet Taste Receptors and Genes Related to Glycolipid Metabolism in Liver

**DOI:** 10.3389/fphar.2022.916603

**Published:** 2022-08-17

**Authors:** Meng-Juan Luo, Ying Wang, Si-Yu Chen, Ze-Min Yang

**Affiliations:** Department of Biochemistry and Molecular Biology, School of Life Sciences and Biopharmaceutics, Guangdong Pharmaceutical University/Guangdong Provincial Key Laboratory of Pharmaceutical Bioactive Substances, Guangzhou, China

**Keywords:** type 2 diabetes mellitus, sweet taste receptors, *Astragalus* polysaccharide, glycolipid metabolism, liver

## Abstract

Sweet taste receptors (STRs) play an important role in glucose metabolism, and type 2 diabetic rats have abnormal expressions of STRs in multiple tissues. *Astragalus* polysaccharides (APS) has shown a significant therapeutic effect on type 2 diabetes mellitus (T2DM), but its mechanism needs to be further clarified. T2DM rat model was induced by intraperitoneal streptozotocin injection and treated with APS for 8 weeks. Daily indicators of experimental rats were observed, and expression levels of STRs and genes related to glycolipid metabolism were determined by real-time quantitative PCR and western blot. The present study revealed that APS alleviated the symptoms of T2DM rats, improved HOMA-IR and promoted insulin secretion. Gene expression analysis found that APS significantly increased the expressions of signaling molecules in STRs pathways, including taste receptor family 1 member 2 (T1R2), α-gustducin (Gα) and transient receptor potential cation channel subfamily member 5 (TRPM5), and reversed the expressions of genes related to glucolipid metabolism, including glucose transporters 2 and 4 (GLUT2 and GLUT4), pyruvate carboxylase (PC), fatty acid synthase (FAS) and acetyl-CoA carboxylase (ACC) in the liver of T2DM rats. However, APS had no influences on the expressions of genes, including glycogen synthase kinase-3 beta (GSK-3β), pyruvate kinase (PK) and phosphoenolpyruvate carboxykinase (PEPCK) in the liver of T2DM rats. These results suggested that the physiological roles of STRs in the liver were involved with glucose transport and metabolism. APS alleviated T2DM rats by activating the STRs pathway, and promoted glucose transport and lipogenesis.

## Introduction

Based on the guidelines of the Chinese Diabetes Society (2020 edition), the nearest epidemiological survey data displayed that diabetes prevalence in China had climbed to 11.2%, among which, type 2 diabetes mellitus (T2DM) patients accounted for more than 90% of diabetics (Society, 2021). T2DM is considered a chronic endocrine and metabolic disease, usually accompanied by vascular function, arteriosclerosis, dyslipidemia, oxidative stress, and hypertension (Bornfeldt and Tabas, 2011; Laakso and Kuusisto, 2014; Galicia-Garcia et al., 2020). Obese people, middle-aged and old people also have more likely to develop the T2DM. Insulin resistance and relatively insufficient insulin secretion are mainly characteristics of the pathophysiology of T2DM (Kerner et al., 2014; Roden and Shulman, 2019); however, it remains unclear in the etiology and pathogenesis of T2DM.

Heredity and diet are two vital pathogenic factors of T2DM, and the occurrence of T2DM is closely related with sugar intake. Sugar sensing is mainly by the sweet taste receptors (STRs) in the oral taste buds, so the expression levels of STRs will directly influence the sugar intake. The STRs in humans and other animals are composed of a heterodimer of taste 1 receptor member 2/3 (T1R2/T1R3), which is part of G protein-coupled receptors (GPCRs) (Nelson et al., 2001; Li et al., 2002; Chaudhari and Roper, 2010). In the oral cavity, the T1R2/T1R3 binds with sweet sugar, activating α-Gustducin (Gα gust) and opening transient receptor potential cation channel sub-family member 5 (TRPM5) *via* a canonical phospholipase C-β/inositol trisphosphate (IP3)-signaling cascade, finally delivering the taste signal by the sensory afferent fiber (Boughter and Munger, 2013; Riccio et al., 2015). Recent research found those extra-oral tissues, including the brain, pancreas, stomach, gut, and liver, were also expressed STRs (Yamamoto and Ishimaru, 2013; Iwatsuki and Nakajima, 2018). These STRs could not deliver the taste signal, but they could sense the levels of sugar in the blood and digestive tract, and then participate in the regulation of metabolism. For example, activated intestinal STRs promoted glucose uptake and release of glucagon-like peptide 1 (GLP-1) by cooperating with the expressions of glucose transporters 2 (GLUT2) and sodium-glucose co-transporter 1 (SGLT-1), while activated pancreatic STRs promoted insulin secretion by cooperating with GLUT2-dependent insulin secretion pathway. Clinical observation found that T2DM patients had impaired taste sensitivity, especially sweet taste (Bustos-Saldana et al., 2009; Wasalathanthri et al., 2014; Yu et al., 2014). Several studies demonstrated that the levels of GLP-1 were decreased in T2DM rats, which were correlated with the impaired intestinal STRs pathway. Our latest research discovered that the deficiency of insulin secretion in T2DM rats was associated with decreased expressions of signaling molecules from the STRs pathway in the pancreas (Yang et al., 2021). Thus, it can be seen, STRs played an important role in metabolism regulation, and T2DM rats might had abnormal expressions of STRs in multiple tissues and organs.

At present, chemical drugs are used as the main treatment way to control T2DM. Although these drugs have good curative effects on control of blood glucose, the effects of preventive are not satisfactory on the occurrence of diabetes complications. In the treatment of diabetes, traditional Chinese herbs have a long history. It emphasizes the significance of the metabolism regulation as a whole in controlling of blood glucose, thereby showing great efficiency on alleviating T2DM and its complications. Among 30 antidiabetic formulas approved by the State Food and Drugs Administrator of China, *Astragalus membranaceus* (Huang qi) is the most frequently used a herb. Moreover, in the treatment of diabetic complications, including nephropathy, neurologic and cardiovascular diseases, *Astragalus membranaceus* is also single herb commonly utilized (Xie et al., 2011). *Astragalus* polysaccharides (APS) was an important active ingredient in the *Astragalus membranaceus* (Fisch.) Bge of a leguminous plant. Previous studies have reported that APS could improve the functions of the digestive system, regulate immunity and antioxidation, and improve the glucose and lipid metabolisms (Fu et al., 2014; Zhang et al., 2019; Zheng et al., 2020). Our previous studies found that APS not only reduced hepatic lipidosis and oxidation-induced damage in hyperlipidemia rats, but also promoted insulin secretion and protected the function of islet *β* cells in T2DM rats (Tang et al., 2017; Chen et al., 2018; Yuan et al., 2018). Although APS was involved with metabolisms of glucose and lipid, its mechanism needed to be further clarified.

Taken together, STRs regulated glucose uptake and glycolipid metabolism, which were the potential targets for the treatment of metabolic disorders. However, liver is a crucial organ of glycolipid metabolism in our body (Rui, 2014). The physiological function of STRs in liver has not been reported, and the relationship between hepatic STRs and T2DM need to be further confirmed. In this study, we observed the expressions of genes, including signaling molecules of STRs pathway, glucose transporters and other genes related with glucolipid metabolism in the liver of T2DM rats, and further explored the function of STRs in the liver and the improvement of APS. This study will provide a theoretical basis for the physiological function of STRs and the exploitation of APS in the treatment of T2DM.

## Materials and Methods

### Chemicals and Reagents

APS was purchased from Xi’an Ruilin Biotechnology Co., Ltd. (batch no. RL20150412, Shanxi, China) with more than 90% purity of polysaccharides from Astragalus membranaceus Bunge, streptozotocin (STZ, Sigma, United States), 10% pelltobarbitalum natricum (batch no. 140418, Beijing solarbio science & technology co., Ltd.), Insulin Detection kit (batch no. CBS-E05070R, CUSABIO biotechnology, Wuhan, China), TRNzol universal Reagent, FastKing gDNA Dispelling RT SuperMix and the Talent qPCR PreMix (SYBR Green) were bought from Tiangen Biotech (Beijing, China), phenylmethylsulfonyl fluoride (PMSF) and 5 × SDS-PAGE Sample Loading Buffer were purchased from Biosharp biochemistry (Guangzhou, China), β-actin, T1R2, Gα and GLUT2 were purchased from Bioss Biotechnology (Beijing, China), Horseradish Peroxidase-conjugated Affinipure Goat Anti-Rabbit IgG (EarthOx Life Sciences, United States).

### Extraction and Identification of Structures of *Astragalus* Polysaccharides

The extraction process of APS was briefly described as follows: Firstly, the crude polysaccharide was extracted with distilled water by the method of ultrasonic wave. Secondly, the polysaccharide extract was condensed and precipitated with ethanol after deproteinization. Finally, APS powder was obtained using the method of freeze-drying.

The total carbohydrate content in APS powder was 90%. Using the method of non-targeted metabolomic analysis based on Liquid Chromatography-Mass Spectrometry (LC-MS), APS was identified 14 highly dependable monosaccharides and oligosaccharides, including D-(+)-Cellobiose, D-Lactose, Gluconic acid, D-Fructose, Maltohexaose, Maltotriose, Maltoheptaose, Maltopentaose, maltotetraose, Apigenin-7-O-neohesperidoside, β-D-Galactopyranose, L-(+)-Arabinose, 4,6,8-Trihydroxy-7-methoxy-3-methyl-3,4-dihydro-1H-isochromen-1-one and Apigenin-8-C-glucoside-2′-rhamnoside. Among them, Maltoheptaose (11%), Gluconic acid (12%), D-Lactose (31%), D-Fructose (13%), D-(+)-Cellobiose (17%), and these five saccharides were accounted for more than 80% (Yang et al., 2021).

### Type 2 Diabetes Mellitus Induction, Administration of *Astragalus* Polysaccharides

Twenty four male Sprague-Dawley (SD) rats with 190–210 g of body weight were provided by the Experimental Animal Center of Guangzhou University of Chinese Medicine [production license number: SCXK (Guangdong) 2018-0034]. Our study strictly acted up to the animal welfare ethics and protection regulations, and the experimental animal ethics committee of Guangdong Pharmaceutical University has authorized it [license number was SYXK (Guangdong) 2017-0125].

After adaptive feeding for 3 days, SD rats were divided into the two groups of control (CON, N = 8) and model (N = 16) at random. Rats in model group and in CON group were fed with high sugar and fat diet and a basial diet for 8 weeks, respectively. Then, 12 h after fasting, rats in model group were intraperitoneally injected streptozotocin solution with a dose of 35 mg/kg (the modeling effect of STZ injection alone is more economical and controlled) (Zhuo et al., 2018), and rats in CON group were injected with citrate buffer in equivalent volume. STZ was freshly dissolved in 0.1 mol/L sodium citrate buffer, pH 4.2–4.5. One week after the STZ injection, orbital blood from the experimental rats were collected to measure fasting blood glucose (FBG). The induction of T2DM rat model was considered successful when FBG value of the T2DM group was more than 11.1 mmol/L accompanied by the symptoms of polydipsia, polyphagia, polyuria and weight loss. Subsequently, T2DM model rats of 16 were divided into the T2DM group (N = 8) and APS group (N = 8) randomly. Drug dose was selected according to our preliminary experimental results and references (Gu et al., 2015; Tang et al., 2017). The APS group of rats were intragastrically administrated with APS at a dose of 700 mg/kg/d (Gu et al., 2015; Tang et al., 2017), while the T2DM and CON groups of rats were intragastrically administered with physiological saline in equivalent volume for 8 weeks. During the administration period, rats in CON group were fed with the basial diet, while rats in T2DM and APS groups were fed with high sugar and fat diet. The recipe of basial diet was as follows: 25% wheat, 25% flour, 25% cornmeal, 8% fish flour, 10% bean power, 2% yeast, 4% bone meal, and 1% refined salt. The recipe of high sugar and fat diet was as follows: 52.2% basial diet, 20% sucrose, 15% lard, 1.3% cholesterol, 10% casein, 0.2% sodium cholate, 0.4% stone powder, 0.4% premix, and 0.6% calcium hydrophosphate. The feeding of rat conditions was as follows: relative humidity of 60%–70%, and room temperature of 22°C–25°C, 12 h light-dark cycle (lights on at 8:00 a.m.).

### Oral Glucose Tolerance Test

The normal and diabetic rats were intragastrically administrated with 20% of glucose after ≥12 h of overnight fast. Orbital blood samples were collected at 0, 30, 60, and 120 min. Blood glucose values were determined using the BS-180 automatic biochemical analyzer.

### Sample Collection and Processing

After fasting 12 h, experimental rats were anaesthetized by intraperitoneal injection, using 10% pelltobarbitalum natricum. Subsequently, the experimental rats were sacrificed by draining blood from abdominal aorta. The liver tissues of experimental rats were removed and then stored in a −80°C freezer after quick freezing in liquid nitrogen. The blood samples from abdominal aorta were placed at 4°C for 2 h, then serum samples were collected by centrifugation at 3, 000 × g for 15 min. Finally, these serum samples were stored at freezer of −80°C.

### The Testing of Fasting Blood Glucose and Insulin

This test was performed in accordance with the method of glucose oxidase using Blood Glucose kit by the BS-180 automatic biochemical analyzer (Shenzhen Mindray Biomedical Electronics Co., Ltd., China). Fasting insulin levels were determined with the method of enzyme-linked immunosorbent assay (ELISA) using an Insulin Detection kit on the Elx-800 microplate reader (BioTek Instruments, Inc., United States). These experiments were carried out according to the manufacturer’s instructions and kit’s protocols.

### RNA Isolation and cDNA Synthesis

Approximately 40 mg of the liver tissues were used to extract total RNA using TRNzol universal Reagent. RNA concentration was quantified by the absorbance at 260 nm on a NanoDrop Lite spectrophotometer (Thermo Fisher Scientific, Inc., United States), and the purity of RNA was assessed by the ratio of the readings at 260 and 280 nm. Total RNA of 2 μg of each sample was reverse transcribed into cDNA using FastKing gDNA Dispelling RT SuperMix on T100™ Thermal Cycler (Bio-Rad, California, United States). The reaction condition: reverse transcription of 42°C for 15 min and enzyme inactivation of 95°C for 3 min.

### Real-Time Quantitative PCR

The RT-qPCR experiment was performed using the Talent qPCR PreMix on CFX Connect TM Real-Time System following strictly the company’s protocol (Bio-Rad, California, United States). The PCR thermal cycle was as follows: Initial denaturation of 95°C for 5 min, followed by 40 cycles of 95°C for 30 s, 56°C for 30 s and 72°C for 45 s, and extension of 72°C for 5 min. All procedures were carried out according the manufacturer’s protocol. The housekeeping gene, β-actin, was used as internal reference gene, and a list of employed primers is presented in Table 1. Relative expressions of interesting genes were quantified by the method of 2^−ΔΔct^ (Livak and Schmittgen, 2001).

**TABLE 1:** The product sizes and sequences of primers used in RT-qPCR.

Gene	Forward primer (5′–3′)	Reverse primer (5′–3′)
β-actin	GGA​GAT​TAC​TGC​CCT​GGC​TCC​TA	GAC​TCA​TCG​TAC​TCC​TGC​TTG​CTG
T1R2	GTC​CGC​CAT​TAC​CGT​GTC​CAA​C	CAC​CAG​CAC​CAC​AAT​CCA​GTT​CC
T1R3	GAG​TCT​GAG​CTG​CCA​CTG​AGT​TG	CTG​GCC​AAT​CTG​TCA​CCA​CCT​CTG
Gα	ACA​GTA​ACA​CGT​TGC​AGT​CCA​TCC	CTG​AGG​CGT​CAT​GTC​ACC​ATC​TTC
TRPM5	TCC​GCC​GTG​TGC​TCT​ACA​GG	GCA​GGA​GAA​TGA​CCA​GCC​AGT​TG
GLUT2	CCA​GCA​CAT​ACG​ACA​CCA​GAC​G	CCA​ACA​TGG​CTT​TGA​TCC​TTC​C
GLUT4	ACT​TAG​GGC​CAG​ATG​AGA​ATG	GTA​AGG​GAA​GAG​AGG​GCT​AAA​G
GSK-3β	CAA​AGC​AGC​TGG​TCC​GAG​G	TCC​ACC​AAC​TGA​TCC​ACA​CCA​C
PK	CGT​GGA​CGA​TGG​GCT​CAT​CT	AGG​TTC​ACG​CCC​TTC​TTG​CT
PEPCK	GTC​CCC​CTT​GTC​TAC​GAA​GC	TGC​ATG​ATG​ACC​TTG​CCC​TTA
PC	AGA​TGC​ACT​TCC​ATC​CCA​AG	CCT​TGG​TCA​CGT​GAA​CCT​TT
FAS	CAG​GAA​CAA​CTC​ATC​CGT​TCT​CT	GGA​CCG​AGT​AAT​GCC​GTT​CA
ACC	TTG​GTG​CTT​ATA​TTG​TGG​ATG​G	ATG​TGC​CGA​GGA​TTG​ATG​G

T1R2/T1R3, taste receptor 1 member 2/3; Gα, α-Gustducin; TRPM5, transient receptor potential cation channel subfamily member 5; GLUT2/GLUT4, glucose transporters 2/4; GSK-3β, glycogen synthase kinase-3 beta; PK, pyruvate kinase; PEPCK, phosphoenolpyruvate carboxykinase; PC, pyruvate carboxylase; FAS, fatty acid synthase; ACC, acetyl-CoA carboxylase.

### Western Blot Analysis

On ice, the liver tissues of 0.1 g were homogenized in RIPA lysis buffer with 1 mmol/L phenylmethylsulfonyl fluoride for 30 min, subsequently, centrifuged at 10,000 × g for 10 min at 4°C to extract the total protein in liver of experimental rat. The concentration of total protein was determined on a NanoDrop Lite spectrophotometer. Total protein (30 μg) was boiled with corresponding 5 × SDS-PAGE Sample Loading Buffer at 100°C for 10 min, and then separated using 6%–12% polyacrylamide gel electrophoresis (SDS-PAGE) on Mini-Protean Tetra Cell (Bio-Rad, United States). Subsequently, the separated proteins were transferred onto polyvinylidene difluoride (PVDF) membranes using Mini Trans-Blot Electrophoretic Transfer Cell (Bio-Rad, United States) and blocked with 5% skim milk prepared with PBST (formula: 1 mL Tween-20 dissolve to 2 L PBS) for 2 h. Subsequently, PVDF membranes with proteins were incubated with primary antibodies for β-actin (dilution, 1:2,000), T1R2 (dilution, 1:2,000), Gα (dilution, 1:1,000) and GLUT2 (dilution, 1:1,000) at 4°C overnight. After washing triplicate by PBST, PVDF membranes with proteins were incubated with the corresponding Horseradish Peroxidase-conjugated Affinipure Goat Anti-Rabbit IgG (dilution, 1:10,000) for 2 h. Finally, after washing triplicate with PBST, the protein blots were stained by ECL luminous solution. By GeneGnome XQR gel imaging systems (Syngene, United Kingdom) to obtain the Protein bands and by ImageJ v1.8.0 software to quantify (National Institutes of Health, United States).

### Statistical Analysis

SPSS 18.0 software (IBM Corporation Inc. United States) was used to Data analysis. The difference among three groups was analyzed by the One-way ANOVA and the difference between two groups was compared by students’ unpaired *t*-test. A value of *p* < 0.05 was considered to indicate statistical significance. All data were presented as the mean ± standard deviation ( $\overset{®}{\chi }$ ± S). GraphPad Prism 7.0 software was used to generate the Figures.

## Results

### Oral Glucose Tolerance Test

**FIGURE 1:**
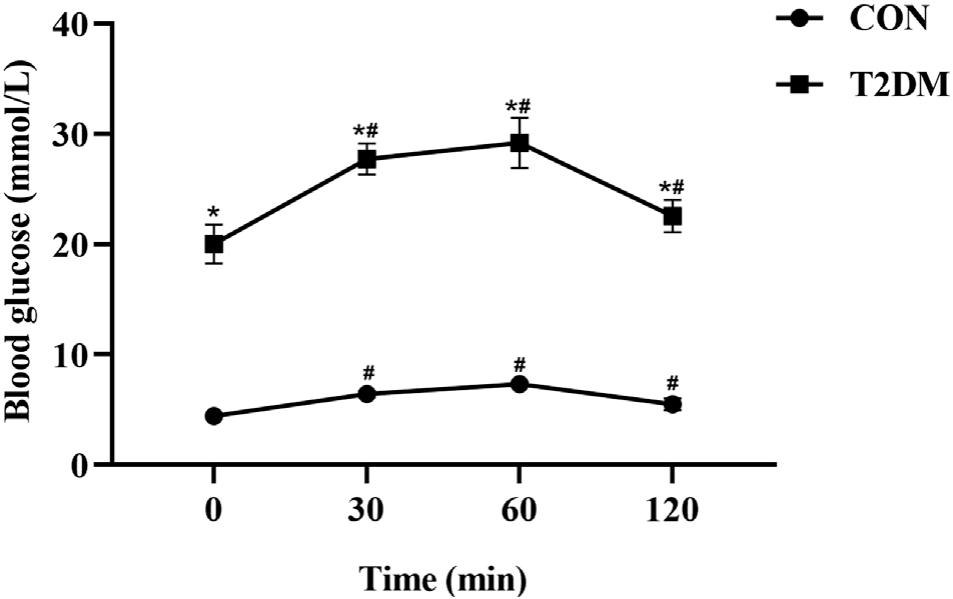
The levels of glucose during the OGTT in the control and T2DM rats. *p < 0.05, compared with rats in CON group; ^#^ *p* < 0.05, compared with blood glucose value in the 0 min.

During the OGTT, the values of blood glucose in CON and T2DM rats were presented in Figure 1. After oral administration of glucose, the blood glucose value of rats in CON and T2DM groups increased significantly (*p* < 0.05), reaching the maximum value at 60 min (32.30 mmol/L, in T2DM group; 7.60 mmol/L, in CON group), and then the blood glucose decreased. During OGTT, the blood glucose value of rats in T2DM group fluctuated significantly and was significantly higher than that of rats in CON group (*p* < 0.05), suggesting that the blood glucose metabolism of rats in T2DM group was abnormal.

### Effects of *Astragalus* Polysaccharides and Daily Indicators of Type 2 Diabetes Mellitus Rats

The daily indicators of experimental rats were observed and the results were shown in Table 2. Rats in the T2DM group had significantly higher intakes of food, water and energy, FBG value and HOMA-IR, and remarkably lower body weight and fasting insulin levels than rats in CON group (*p* < 0.05). After APS administration for 8 weeks, rats in APS group showed significantly lower intakes of food, water and energy, FBG value and HOMA-IR, and higher fasting insulin levels and body weight compared with T2DM rats (*p* < 0.05).

**TABLE 2:** Daily indicators of experimental rats.

	N	Body weight (g)	Food intake (g)	Water intake (mL)	Energy intake (Kcal)	FBG (mmol/L)	FINS (μU/mL)	HOMA-IR
CON	8	575.78 ± 33.25*	613.28 ± 11.31*	1034 ± 59.45*	1901.17 ± 35.07*	5.03 ± 0.75*	35.90 ± 11.97*	7.05 ± 1.84*
T2DM	8	370.30 ± 27.36	807.64 ± 13.96	2030 ± 24.49	3820.14 ± 18.73	22.29 ± 2.15	21.54 ± 2.39	23.57 ± 3.65
APS	8	433.20 ± 15.15*^#^	627.98 ± 10.23*^#^	1198 ± 57.41*^#^	2970.35 ± 44.67*^#^	12.03 ± 0.82*^#^	35.78 ± 11.49*	15.87 ± 2.45*

*p < 0.05, compared with rats in T2DM group.

^#^p < 0.05, compared with rats in CON group.

### Effects of *Astragalus* Polysaccharides on mRNA Expressions of Signaling Molecules in Sweet Taste Receptors Pathway in the Liver of Type 2 Diabetes Mellitus Rats

**FIGURE 2:**
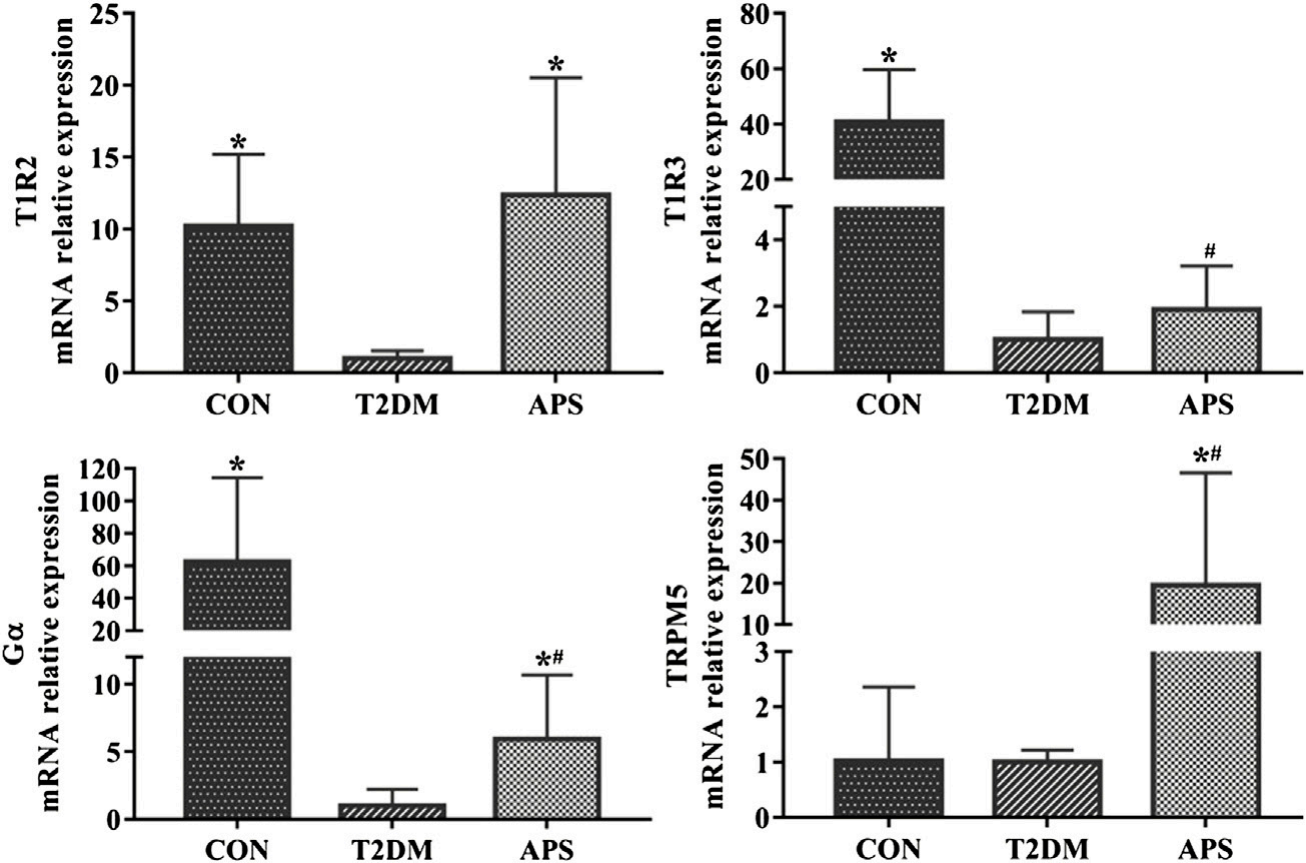
mRNA expressions of signaling molecules in STRs pathway in liver of experimental rats. *p < 0.05, compared with rats in T2DM group; ^#^ *p* < 0.05, compared with rats in CON group.

In order to evaluating the effect of APS on STRs in liver, we measured mRNA expression levels of signaling molecules in STRs pathway in the liver of experimental rats (Figure 2). The results showed that the mRNA expressions of T1R2, T1R3, and Gα were significantly lower in the liver of rats in T2DM group than those of rats in the CON group (*p* < 0.05). After APS treatment for 8 weeks, the mRNA expression levels of T1R2, Gα and TRPM5 in the liver of rats in the APS group significantly increased compared with those of rats in the T2DM group (*p* < 0.05), but no significant differences in the mRNA expression levels of T1R3 was found between rats in T2DM and APS groups.

### Effects of *Astragalus* Polysaccharides on mRNA Expressions of Genes Related to Glucose and Lipid Metabolism in the Liver of Type 2 Diabetes Mellitus Rats

**FIGURE 3:**
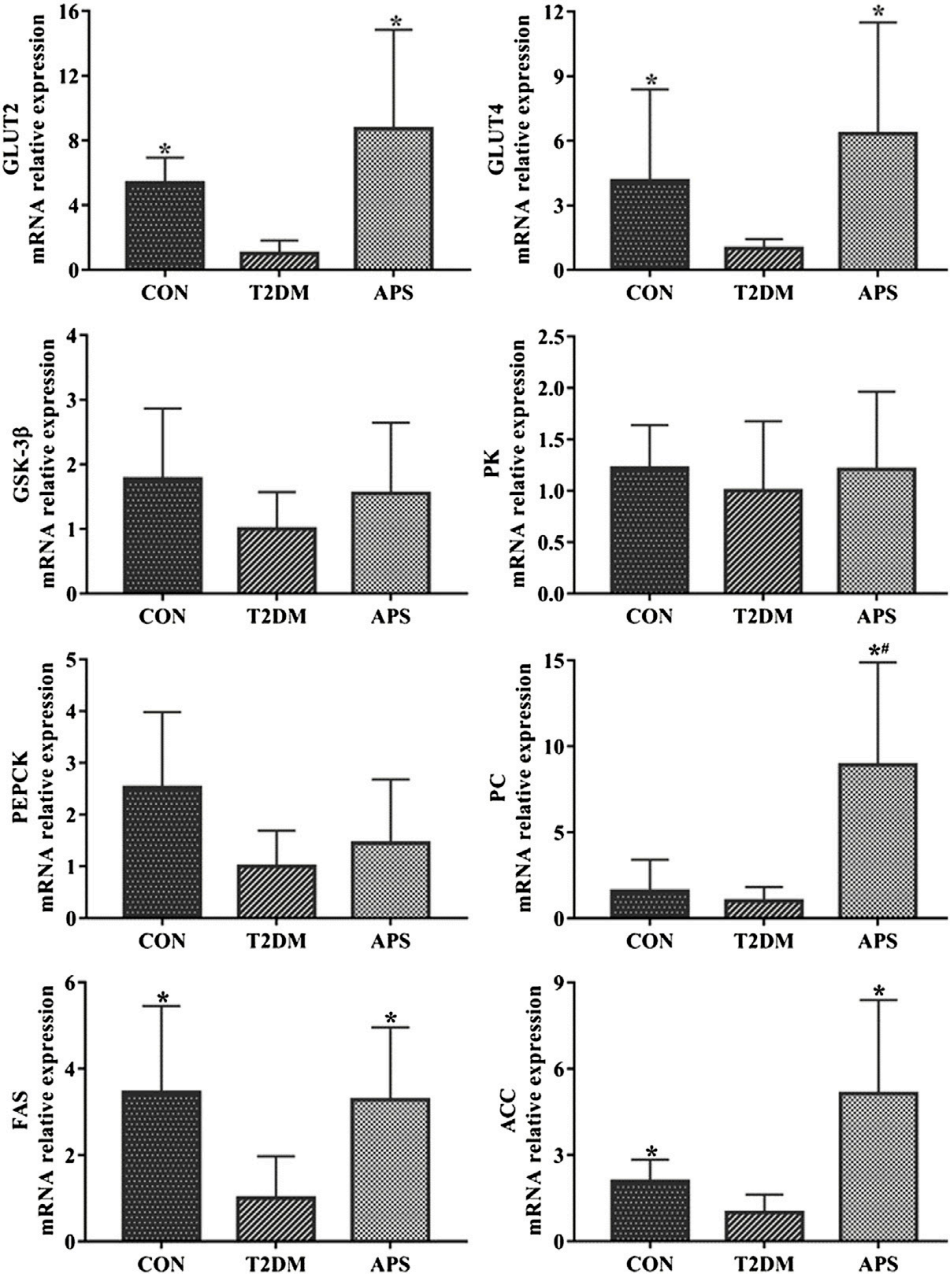
mRNA expressions of genes related to glucose and lipid metabolism in liver of experimental rats. *p < 0.05, compared with rats in T2DM group; ^#^ *p* < 0.05, compared with rats in CON group.

In order to explore the physiological function of STRs in the liver and the effect of APS on hepatic glucose and lipid metabolism, we measured mRNA expression levels of genes related to glucose and lipid metabolism in liver of experimental rats (Figure 3). The results showed that the mRNA expressions of GLUT2, GLUT4, FAS, and ACC were considerably lower in the liver of rats in T2DM group than those of rats in CON group (*p* < 0.05). After APS treatment for 8 weeks, the mRNA expression levels of GLUT2, GLUT4, PC, FAS, and ACC in the liver of rats in APS group significantly increased compared with those of rats in T2DM group (*p* < 0.05). No differences in the mRNA expressions of GSK-3β, PK and PEPCK in the liver were found among rats in CON, T2DM, and APS groups.

### Effects of *Astragalus* Polysaccharides on protein expressions of T1R2, Gα, and GLUT2 in the liver of Type 2 Diabetes Mellitus Rats

**FIGURE 4:**
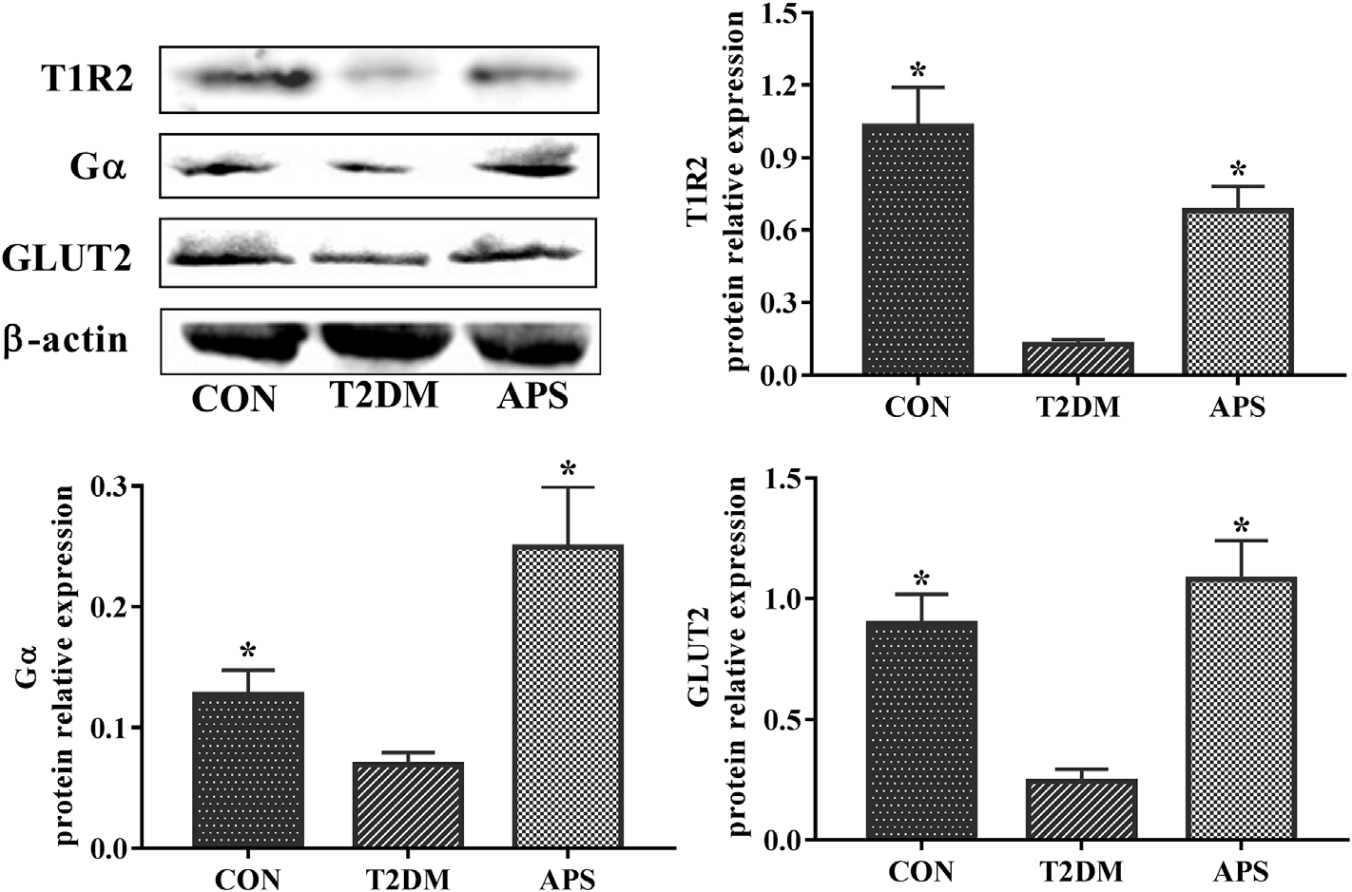
Protein expressions of T1R2, Gα and GLUT2 in liver of experimental rats. *p < 0.05, compared with rats in T2DM group; ^#^ *p* < 0.05, compared with rats in CON group.

To verify the results in mRNA expressions of gene, which was determined by the method of RT-qPCR, we determined the protein expressions of T1R2, Gα, and GLUT2 in liver of experimental rats by the method of western blot (Figure 4). The results showed that the protein expression levels of T1R2, Gα, and GLUT2 significantly reduced in the liver of rats in T2DM group compared with those of rats in CON group (*p* < 0.05). After APS treatment for 8 weeks, the protein expression levels of T1R2, Gα, and GLUT2 obviously increased in the liver of rats in APS group compared with those of rats in T2DM group (*p* < 0.05). This results in protein expression supported effectively the data in mRNA expression.

## Discussion

The present study revealed that APS alleviated the symptoms of T2DM rats. The mechanism might be promote uptake of glucose and fatty acid synthesis in the liver, by increasing gene expressions of signaling molecules in the STRs pathway, glucose transporters and fatty acid synthetases in the liver of T2DM rats. These findings provided data proof for understanding physiological functions of hepatic STRs and therapeutic effect of APS on T2DM.

STRs are composed of a heterodimer of T1R2 and T1R3, which belongs to G protein-coupled receptors family (Wang et al., 2018). Some research found that extra-oral tissues, including the stomach, liver, brain, pancreas, gut, and fat, were also expressed STRs (Yamamoto and Ishimaru, 2013; Lee and Owyang, 2017; Iwatsuki and Nakajima, 2018; Lee et al., 2019). These STRs could not deliver the taste signal, but they could sense the levels of sugar in the blood and digestive tract, and then participate in regulation of metabolism. The physiological function of STRs in the oral cavity and intestine has been discovered in many researches. Among these researches, the oral STRs were considered to sense natural sugars and artificial sweeteners, regulating nutrients intake, while the intestinal STRs were verified to sense sweet carbohydrates from digestive tract, coordinating with glucose transporters to promote glucose uptake, glucagon-like peptide 1 (GLP-1) secretion and postprandial insulin secretion. The STRs in pancreas and brain may be able to sense the level of blood glucose and regulate glucose metabolism. Based on the previous studies that we found, the pancreatic STRs regulated insulin secretion by collaborating with glucose transporters (Yang et al., 2021). However, few studies reported the physiological function of STRs in the liver, which is an important organ of metabolism. Clinical research found that T2DM patients had less sensitivity to sweetness, which could lead to a preference for sweet-tasting foods, thereby exacerbating hyperglycemia (Gondivkar et al., 2009; Dias et al., 2016; Pugnaloni et al., 2020). Animal model studies from our group found the decreased expressions of signaling molecules in the STRs pathway in the intestine of the T2DM and hyperlipidemia rats compared with these of control rats (Wang et al., 2019; Yang et al., 2021). Similar results were also reported by Feng et al. (2017). Moreover, our previous study found that insufficient insulin secretion in the T2DM rats was closely related to the decreased expressions of signaling molecules in the STRs pathway in the pancreas (Yang et al., 2021). Taken together, there might be the impaired expressions of STRs in several tissues of the T2DM, and STRs could be used as a potential target for the treatment of T2DM. In the present study, we investigated the STRs pathway in the liver of T2DM rats. Our results found that the expressions of STRs pathway signaling molecules, including T1R2/T1R3 and Gα, in the liver of T2DM rats remarkably decreased when compared with the control rats. These findings suggested that the impaired STRs pathway might be related to the disorder of glucose and lipid metabolism in the liver of T2DM rats.

GLUT2 is a bidirectional glucose transporter and expressed in the liver, kidney and pancreas, GLUT2 mediates the bidirectional glucose transport and efflux of glucose from the hepatocytes after insulin stimulation (Narasimhan et al., 2015). GLUT2 in the hepatocytes has low affinity and high transport efficiency for glucose, and it is sensitive to reflect the high glucose environment, which is helpful for the liver to play a key role in regulating whole-body glucose homeostasis (Thorens, 2015). GLUT4 is a major transporter of glucose removal from the circulation and highly expressed in the adipose tissue and skeletal muscle. The expression and transport of GLUT4 are regulated by insulin, which is critical to maintain the blood glucose balance. As liver is one of the main target organs of insulin, GLUT4 plays a rate limiting role in glucose uptake in the liver. Pyruvate kinase (PK) is a part of the key enzymes in the glycolytic pathway. Pyruvate carboxylase (PC) and phosphoenolpyruvate carboxykinase (PEPCK) are the two key enzymes in the gluconeogenesis pathway, the expression levels of the three genes of PK, PC and PEPCK regulate the oxidation and synthesis of glucose. Glycogen synthase kinase-3 beta (GSK-3β) reduces the synthesis of hepatic glycogen by inhibiting the activity of glycogen synthase through phosphorylation. Fatty acid synthase (FAS) and acetyl-CoA carboxylase (ACC) are two key enzymes in fatty acid synthesis. The expression levels of the three genes of GSK-3β, FAS, and ACC determine whether excessive glucose in the hepatocytes is converted into glycogen or fat? Some researchers found that glucose entered the hepatocytes via GLUTs, and then it rapidly transformed into glucose 6-phosphate (G-6-P) by glucokinase (GK), further maintained a low glucose concentration in the cells, so that the glucose was continued to flow into hepatocytes from the blood and stored in the hepatocytes. Moreover, the increased glucose in the hepatocytes was produced xylulose 5-phosphate via the pentose phosphate pathway, further activated protein phosphatase 2 (PP2). Activated PP2 was dephosphorylated the carbohydrate response element binding protein (ChREBP) (Kabashima et al., 2003; Dentin et al., 2005; Dentin et al., 2006), which had resulted in translocation of ChREBP into the nucleus and activated the transcription of the lipogenic genes such as ACC and FAS, it is promoted fat synthesis in hepatocytes (Thorens, 2015). T2DM is characterized by hyperglycemia and accompanied with metabolism disorders of lipid. Clinical observation found that T2DM patients and animal models showed the damage of glucose transport, enhancement of gluconeogenesis, and dysfunction of glycoxidation, and were accompanied by the increased degradation of fat and protein. At present, sodium-dependent glucose transporters 2 (SGLT2) inhibitor has been widely used in the treatment of T2DM and GLUT2 has also become an important target for diabetic treatment. Furthermore, the abnormal expression and transport of GLUT4 is one of the main manifestations of insulin resistance or T2DM. In addition, Gao et al. (2021) revealed that ACC aggravated the insulin resistance and metabolism disorders of the glucose and lipid by inhibiting the AMPK/ACC pathway. The present study found that the expressions of GLUT2, GLUT4, FAS, and ACC significantly decreased in the liver of T2DM rats compared with the control rats, while no significant differences in the expressions of genes involved in glucose metabolism, including PK, PC, PEPCK, and GSK-3β were found between rats in the CON and T2DM groups. These results suggested that T2DM rats were mainly manifested as the impairment of glucose transport and deficiency of fatty acid synthesis in the liver, and the disorder of glucose metabolism was not obvious.

*Astragalus* polysaccharides (APS) is a vital active ingredient of *Astragalus* membranaceus. Pharmacological studies revealed that APS could effectively regulate the function of immune and improve insulin resistance. Furthermore, APS has shown a good application prospect for the comprehensive prevention and treatment of diabetes, hyperlipidemia and complications. Our previous studies found that APS improved glucose and lipid metabolism in the T2DM and hyperlipidemia rats, protected the functions of pancreas and liver (Chen et al., 2018; Yuan et al., 2018). Our further studies revealed that APS increased the expressions of GLUT2 and signaling molecules in the STRs pathway in the intestinal and pancreatic of T2DM rats, promoted intestinal glucose uptake and GLP-1 secretion, coordinated pancreatic insulin secretion and improved the symptoms of T2DM (Wang et al., 2019; Yang et al., 2021). ZOU and ZHANG reported that APS increased the expression of GLUT4 in adipose tissue and promoted the translocation of GLUT4 from microbubbles to the plasma membrane in skeletal muscle, improved the insulin resistance of T2DM rats (Zou et al., 2009; Zhang et al., 2018). In addition, several researchers found that APS enhanced insulin sensitivity and ameliorated hepatic steatosis by regulating the expression of GSK3β (Mao et al., 2006; Mao and Ou-Yang, 2007), although these results were contradictory. Mao et al. (2019) found that APS reversed the over-expression of PEPCK in the insulin resistant model of HepG2 cells. The present study confirmed that APS alleviated the symptoms of T2DM rats, increased insulin levels and improved insulin HOMA-IR. Furthermore, the gene expression analysis showed that APS significantly increased the expression levels of signal molecules (T1R2, Gα, TRPM5) in the STRs pathway, glucose transporters of GLUT2 and GLUT4, and fatty synthesis genes of FAS and ACC in the liver of T2DM rats. However, APS had no obvious effects on the expression levels of glucose metabolism related genes of GSK-3β, PC, PK, and PEPCK. These results suggested that APS alleviated the symptoms of T2DM rats by ameliorating the STRs pathway and the expression of glucose transporters in the liver, thereby promoting glucose uptake and fatty synthesis.

## Conclusion

**FIGURE 5:**
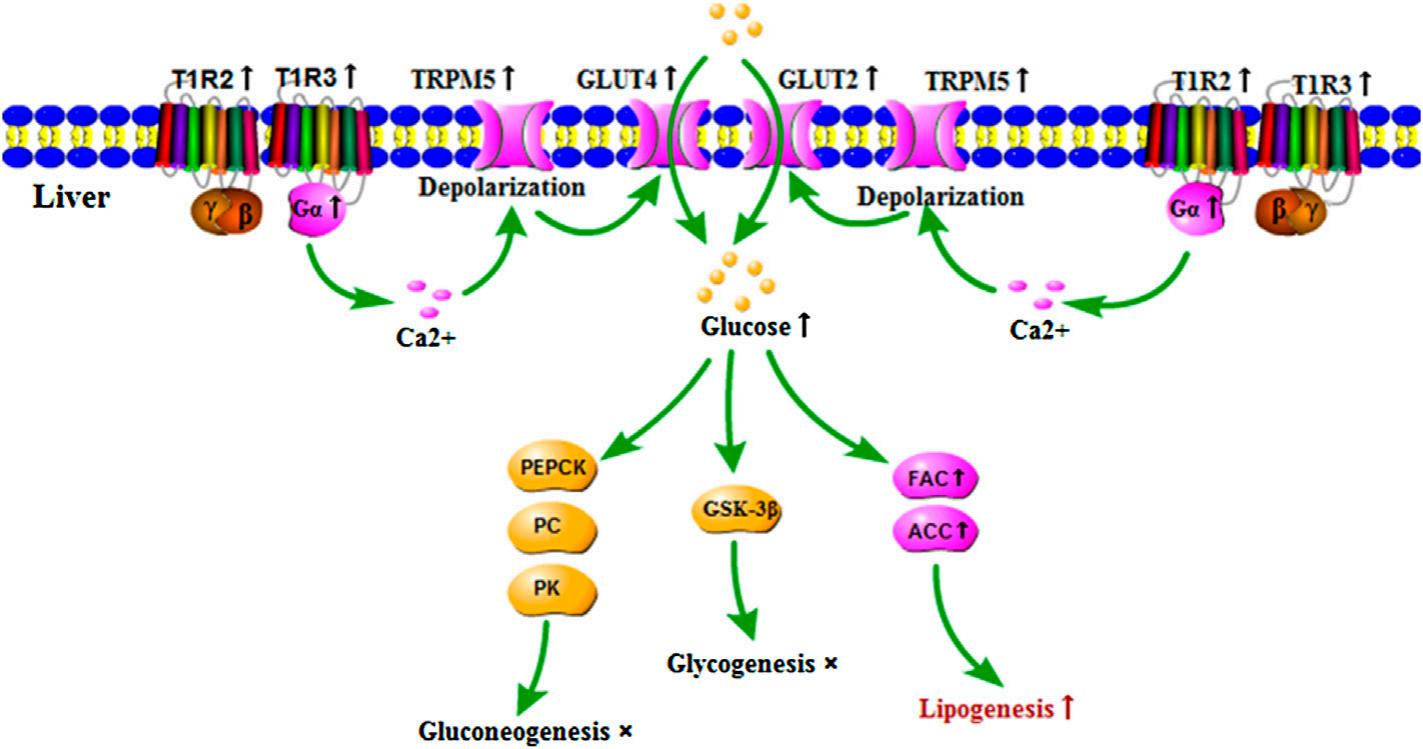
Regulatory role of APS on expressions of signaling molecules in STRs pathway and genes of glucose and lipid metabolism in the liver of T2DM rats. ↑: indicated that APS up-regulated gene expressions and biological process of T2DM rats, ×: indicated that no significant influence of APS on biological process of T2DM rats.

In summary, the current study demonstrated that APS reversed the attenuated expression of signaling molecules in the STRs pathway, glucose transporters, and fatty synthesis genes in the liver of T2DM rats and promoted the hepatocyte glucose uptake and lipogenesis, thereby alleviating the symptoms of T2DM rats. Nevertheless, APS had no significant effect on the glucose metabolism in the present study (Figure 5). These findings provided a beneficial attempt for the functional study of STRs in the liver and the development of APS in the treatment of T2DM. However, the relationship between STRs and glucose transporters in the liver needs to be further confirmed.

## Data Availability

The raw data supporting the conclusion of this article will be made available by the authors, without undue reservation.
